# Plant-Derived Polyphenols as Nrf2 Activators to Counteract Oxidative Stress and Intestinal Toxicity Induced by Deoxynivalenol in Swine: An Emerging Research Direction

**DOI:** 10.3390/antiox11122379

**Published:** 2022-12-01

**Authors:** Jun Chen, Zhouyin Huang, Xuehai Cao, Xingping Chen, Tiande Zou, Jinming You

**Affiliations:** Jiangxi Province Key Laboratory of Animal Nutrition, Jiangxi Province Key Innovation Center of Integration in Production and Education for High-Quality and Safe Livestock and Poultry, Jiangxi Agricultural University, Nanchang 330045, China

**Keywords:** antioxidants, deoxynivalenol, intestinal toxicity, oxidative stress, plant-derived polyphenols, swine

## Abstract

The contamination of deoxynivalenol (DON) in feed is a global problem, which seriously threatens the productivity efficiency and welfare of farm animals and the food security of humans. Pig is the most sensitive species to DON, and is readily exposed to DON through its grain-enriched diet. The intestine serves as the first biological barrier to ingested mycotoxin, and is, therefore, the first target of DON. In the past decade, a growing amount of attention has been paid to plant-derived polyphenols as functional compounds against DON-induced oxidative stress and intestinal toxicity in pigs. In this review, we systematically updated the latest research progress in plant polyphenols detoxifying DON-induced intestinal toxicity in swine. We also discussed the potential underlying mechanism of action of polyphenols as Nrf2 activators in protecting against DON-induced enterotoxicity of swine. The output of this update points out an emerging research direction, as polyphenols have great potential to be developed as feed additives for swine to counteract DON-induced oxidative stress and intestinal toxicity.

## 1. Introduction

In the modern swine industry, feed costs account for 60~70% of the total costs of pork production [1], and mycotoxin contamination in feed is a serious issue, especially deoxynivalenol (DON). The contamination of DON in feed causes huge economic losses annually for the global swine industry [2,3]. Mycotoxin DON contamination in feedstuffs is a worldwide, concerning issue, and is of great threat to the production efficiency and well-being of farm animals, as well as the food security of humans. Gruber-Dorninger et al. (2019) conducted a 10-year survey regarding global mycotoxin occurrence in feed [4]. The authors documented that at least one kind of mycotoxin was detected in 88% of 74,821 samples from 100 countries, and a positive rate of 84.8% was determined in 13,232 feed samples in East Asia [4]. According to another survey performed in China, DON is the most abundant mycotoxin in feed samples among mycotoxins, with a content of up to 4 mg/kg [5]. DON is a type B trichothecene mycotoxin, which is mainly produced by *Fusarium culmorum* and *Fusarium graminearum,* and contaminates common feed grains including wheat, maize, barley, and oats [6]. Because the complete diets of pigs are enriched in grain feedstuffs, pigs are readily exposed to DON through their grain-enriched diet [7,8]. Moreover, the pig is the most sensitive species to DON; the DON sensitivity rank order is pig > mouse > rats > poultry ≈ ruminants [9]. The upper limit level of DON in pig feed is 0.9 mg/kg, which is only 1/5 of the upper limit of DON in poultry feed [10]. According to the aforementioned reasons, DON contamination in feed is most harmful to pigs. The intestine has been demonstrated as one of the critical targets of food-borne mycotoxins [11,12]. The intestine is the first and foremost exposed target following contaminated food ingestion [13], and serves as the first biological barrier to ingested mycotoxins. It is, therefore, the first target of DON [14]. When pigs consume DON-contaminated feed, the intestine is exposed to high concentrations of DON [15]. The clinical symptoms of DON exposure in pigs are anorexia, vomiting, and growth performance reduction [16]. Those clinical signs (i.e., vomiting, etc.) are observed only after a high intake of DON, and are considered signs of acute toxicosis. However, intestinal disturbances are the common signs observed at a level which is common in daily practice. Most recently, the induction of an oxidative stress response due to DON exposure has been documented to be one of the critical mechanisms of action for its toxicity in swine [16,17]. The key role of oxidative stress in DON-induced enterotoxicity is mainly attributed to (1) the overgeneration of reactive oxygen species (ROS) by DON exposure, followed by antioxidant/prooxidant imbalance (oxidative damage, including proteins, DNA, and lipids); (2) the reduction in antioxidant ability by DON exposure (such as glutathione peroxidase (GSH-Px) and (superoxide dismutase) SOD); and (3) the activation of related pathways induced by oxidative stress (such as mitogen-activated protein kinases (MAPKs) and nuclear factor kappa-B (NF-κB)). It has been demonstrated that oxidative stress is a vital determining factor responsible for DON-induced intestinal toxicity in animals, and the role of oxidative stress caused by DON intake should be considered as a key mechanism for DON toxicity [18]. In this regard, natural antioxidants should be the best candidates to counteract gut toxicity caused by DON-contaminated feed in pigs. Most recently, increasing attention has been focused on natural plant-derived polyphenols to ameliorate gut toxicity by DON exposure in pigs. However, to the best of our knowledge, no reviews are available regarding polyphenol intervention strategies to counteract intestinal toxicity caused by DON. The scope of this review is primarily intended to summarize polyphenol intervention strategies to counteract the intestinal toxicity caused by DON in pigs.

## 2. The Physicochemical Characteristics of DON

**Figure 1:**
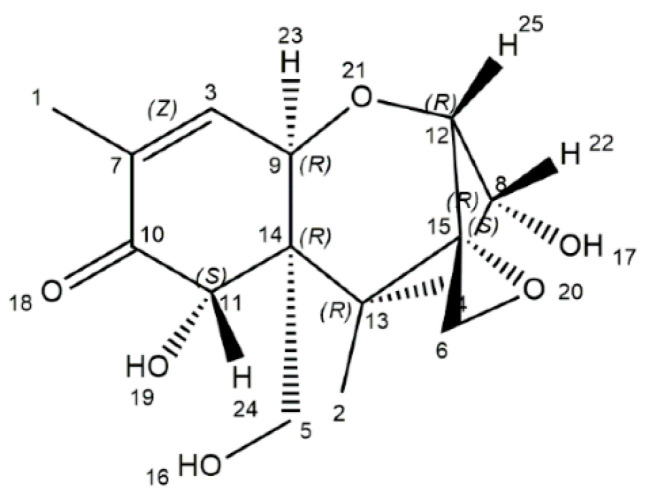
The chemical structure of deoxynivalenol [19].

DON, a naturally occurring mycotoxin, is predominantly produced by *Fusarium culmorum* and *Fusarium graminearum* [18]. The DON is characterized by a chemical formula of C_15_H_20_O_6_ (molecular weight: 296.32), and its chemical name is 12,3-epoxy-3α,7α,15-trihydroxytrichothec-9-en-8-on (Figure 1) [19]. Structurally, DON includes three free hydroxy groups (-OH), which are greatly related to its toxicity [19]. Physically, DON is a fine, colorless needle (melting point: 151–153 °C; boiling point: 543.9 ± 50.0 °C; flash point: 206.9 ± 2.5 °C), and is soluble in polar organic solvents and water [20]. DON is a heat-stable mycotoxin, which is quite stable within 170–350 °C, as evidenced by the absence of a decreased concentration of DON after 30 min treatment at 170 °C. Those physicochemical characteristics of DON are of great threat to humans and farm animals [7,8].

## 3. DON-Induced Enterotoxicity in Swine

Pigs, the most sensitive species to DON, are often at high risk of exposure to DON because corn and wheat are common high-proportion ingredients in their formulated feed [21]. The intestine has been authenticated as one of the most crucial targets of DON exposure [11,12]. DON exposure in pigs is demonstrated to result in intestinal toxicity, including compromised effects on intestinal nutrient absorption [6,22,23,24,25], intestinal morphology, intestinal barrier function, intestinal oxidative stress, intestinal inflammation, and gut microbiota [26]. Interestingly, there is a growing body of evidence that oxidative stress is one of the most crucial underlying mechanisms of action in the intestinal toxicity of DON in swine [16,17]. Oxidative stress is a phenomenon in which the overproduction of ROS exceeds the ROS-scavenging ability or antioxidant capacity of cells [18]. In both in vivo and in vitro porcine studies, DON exposure has been demonstrated to generate free radicals, including ROSs that induce intestinal oxidative damage, which results in alterations in membrane integrity, cellular redox signaling, and changes in the antioxidant status of the intestinal cells [9,17,27,28]. Normally, DON exposure compromises the physiological functions of mitochondria and results in the overproduction of free radicals (including ROS), which further induce oxidative damage, including lipid peroxidation, DNA injury, and protein oxidation [18]. Additionally, DON intake reduces antioxidant enzymes, such as SOD and GSH-Px [16,29]. Some signaling pathways, including MAPKs, are subsequently induced by oxidative stress, and caspase-mediated apoptosis pathways are activated as well [14]. In addition, NF-κB is activated following oxidative stress induced by DON, which further drives its downstream expression of pro-inflammatory genes (such as IL-8, IL-1β, IL-6, IFN-γ, and TNF-α) [29]. These best explain that DON exposure induces oxidative stress, inflammation, and even apoptosis of the intestine in swine. In short, oxidative stress is a critical underlying mechanism of DON-induced enterotoxicity in pigs. Because a large number of works of literature have reported the toxic effects of DON on the porcine intestine, there is a perfect review summarizing the negative effects of DON on the porcine intestine [16]. Therefore, this review will not expand on DON enterotoxicity in piglets, but will systemically update the protective effects and underlying mechanisms of plant-derived polyphenols in alleviating DON-induced oxidative stress and intestinal toxicity in swine.

## 4. Plant-Derived Polyphenol Application to Counteract Oxidative Stress and Intestinal Toxicity Induced by Deoxynivalenol in Swine

It has been reported that oxidative stress is a crucial factor contributing to DON-caused intestinal toxicity in animals [30], and the role of oxidative stress induced by DON exposure should be regarded as one of the key mechanisms for its toxicity [18]. Alleviating oxidative stress would be an effective way to attenuate the toxic effects of DON, thereby reducing its damage to farm animals [17]. In this regard, antioxidants should be the best candidates to counteract oxidative stress and gut toxicity caused by DON-contaminated feed. Polyphenols are a large group of plant secondary metabolites with strong antioxidant abilities, which is attributed to their special chemical structure (aromatic rings with multiple hydrogen hydroxyl groups) [27]. Recently, increasing attention has been focused on polyphenols to detoxify gut toxicity by DON exposure [10,29,31,32,33]. Thus, this review mainly focuses on polyphenol intervention strategies to counteract DON-induced gut toxicity. An increasing number of in vivo and in vitro studies have been collected and described as evidence compatible with a role for oxidative stress in DON-induced intestinal toxicity. For these reasons, multiple studies have been performed using polyphenols to attempt to counteract the adverse effects of oxygen radicals generated under DON treatment. The summary of plant-derived polyphenols counteracting DON-induced enterotoxicity in swine, both in vivo or in vitro, is presented in Table 1.

### 4.1. Resveratrol

Resveratrol (3,5,4′-trihydroxystilbene, C_14_H_12_O_3_) is a plant-derived polyphenolic antioxidant that is abundant in grape skin and red wine [32]. Most recently, resveratrol has been reported to possess efficient antioxidant activity and counteract DON-causing intestinal toxicity in pigs, in both in vivo and in vitro studies. In a 2 × 2 factorial experimental feeding trial, Qiu et al. (2021) reported the beneficial effects of dietary 300 mg/kg resveratrol supplementation on intestinal oxidative injury and inflammation caused by 3.8 mg/kg DON challenge in weaned piglets [31]. The authors found that feeding 300 mg/kg resveratrol for 28 days significantly increased the antioxidant capacity and decreased oxidative stress in the intestines of weaned piglets, while also markedly abrogating their increased intestinal cell apoptosis [31]. In a 28-day feeding trial, Hong et al. (2022) demonstrated that dietary 300 mg/kg resveratrol supplementation effectively alleviated the 2.65 mg/kg DON-induced oxidative stress injury of weaned piglets, and decreased the DON-causing impairment of mitochondria and intestinal morphology of piglets [21]. Specifically, compared with the DON group, piglets from the DON + resveratrol group had higher SOD activity, total antioxidant capacity (T-AOC), mitochondrial membrane potential in the jejunum, as well as decreased malondialdehyde (MDA) content and mitochondria reactive oxygen species (ROS) in the jejunum [21]. Consistently, Qiu et al. (2022) also found that the dietary addition of resveratrol improved intestinal oxidative damage, inflammation, and intestinal microbiota of piglets fed DON contaminated diet [40]. In a study using the IPEC-J2 cell model, Ling et al. (2016) reported that an addition of 50 μM resveratrol protected against 4 µM DON-induced intestinal barrier dysfunction and bacterial translocation [32]. Therefore, resveratrol, as a potent antioxidant agent, could be added as a novel dietary strategy to relieve DON-induced intestinal toxicity in pigs [32]. In another in vitro study using IPEC-J2 cells, Yang et al. (2019) also found that pretreatment with 15 μM resveratrol protected IPEC-J2 cells from cell toxicity by 0.5 μg/mL DON via activating the nuclear factor erythroid 2-like 2 (Nrf2) signaling pathway, and Nrf2 siRNA knockdown abrogated the protective effects of resveratrol on DON-caused intestinal cytotoxicity [10]. The results of Yang et al. (2019) revealed that Nrf2 signaling pathway-mediated antioxidant pathway has quite an important role in the protection of resveratrol against DON toxicity. Apart from the Nrf2 signaling pathway, mitophagy was reported to be another potential mechanism of resveratrol restoring intestinal injury caused by DON challenge in piglets [34]. Using both in vivo and in vitro models, Huang et al. (2022) demonstrated that resveratrol ameliorated the intestinal injury caused by DON through mitophagy in weaned piglets [34]. The authors found that diet contaminated with 3.8 mg/kg DON significantly upregulated the mRNA expression abundance of mitophagy-related genes, while dietary supplementation with 300 mg/kg resveratrol downregulated those parameters in the ileum of piglets. Furthermore, the authors found that DON markedly reduced mitochondrial respiration and nicotinamide adenine dinucleotide (NAD) and total adenosine triphosphate (ATP) of IPEC-J2 cells, while resveratrol supplementation increased the rate of spare respiratory capacity, NAD, and total ATP [34]. These findings indicate that oxidative stress is a crucial factor in DON cytotoxicity, and supplementation with resveratrol can counteract DON cytotoxicity in pigs.

### 4.2. Oxyresveratrol

Oxyresveratrol (trans-3,5,2′,4′-tetrahydroxystilbene, C_14_H_12_O_4_) is one kind of resveratrol analog, which is characterized by an additional hydroxyl group bearing on the aromatic ring in comparison to resveratrol [41]. Oxyresveratrol is more water-soluble than resveratrol. Similarly to resveratrol, oxyresveratrol has robust antioxidant, anti-inflammatory, anti-obesity, and antiviral biological properties [41]. In recent years, oxyresveratrol has been demonstrated to have beneficial regulatory effects on intestinal inflammation and intestinal diseases [42,43]. In a Caco-2 model, oxyresveratrol treatment markedly reduced monolayer permeability by upregulating the gene and protein expression of ZO-1, Claudin-1, and Occludin, which are involved in protein kinase C (PKC) and mitogen-activated protein kinase (MAPK) pathways [44]. Oxyresveratrol has also been reported to alleviate colitis in rats by inhibiting intestinal inflammation [45]. Oxyresveratrol treatment significantly downregulated the gene expression of pro-inflammatory factors (IL-6, IL-1β, and TNF-α), but upregulated the gene expression of anti-inflammatory factors (IL-10). Moreover, the expression of COX-2 and iNOS was inhibited by oxyresveratrol supplementation [45]. Similarly, Ramulus mori ethanol extract, enriched in oxyresveratrol, alleviated acute colitis by inhibiting intestinal inflammation and promoting mucin production in a mouse model as well as in a LS 174T goblet cell line model [46]. In pig production, oxyresveratrol has been documented to be utilized for inhibiting African swine fever virus replication [47]. Wan et al. (2018) noted that treatment with 25 μM oxyresveratrol for 12 h protected IPEC-J2 from DON-caused intestinal barrier dysfunction, as suggested by the increased TEER and protein expression of Claudin-4 [35]. The c-Jun N-terminal kinase (JNK) pathway may be involved in this protective process, because the JNK phosphorylation was inhibited by oxyresveratrol supplementation [35]. Herein, oxyresveratrol is a promising candidate to protect the porcine intestine against DON exposure, and further research is needed to investigate the protective effects of oxyresveratrol on pig intestines in vivo studies.

### 4.3. Baicalin

Baicalin (C_21_H_18_O_11_) is a natural flavonoid derived from Scutellaria baicalensis Georgi, which is one kind of traditional Chinese medicine [48]. Baicalin is characterized by numerous pharmacological properties, including antioxidant, anti-inflammatory, anticancer, and antiviral functions, for protecting intestinal health [33]. In a 14-day feeding study, 320 weaned piglets were allotted to four dietary treatments using a 2 × 2 factorial experimental design. Factor 1 was dietary DON level (0 vs. 4 mg/kg), and factor 2 was dietary baicalin level (0 vs. 0.1%) [33]. The results show that baicalin supplementation alleviated the adverse effects of DON-induced oxidative damage and intestinal inflammation of piglets [33]. Specifically, baicalin supplementation augmented serum antioxidant capacity (increased T-AOC, glutathione (GSH) content, SOD activity, and GSH-Px activity; decreased MDA content), and downregulated the genes’ expression of pro-inflammatory cytokines in intestinal tissues (IL-8, IL-6, IFN-γ, and TNF-α in the jejunum; IL-8, IL-1β, IFN-γ, and TNF-α in the ileum) in piglets fed a DON-contaminated diet [33]. Moreover, baicalin supplementation reversed the impairment of intestinal morphology in piglets fed a DON-contaminated diet, including villus height, crypt depth, and villus height/crypt depth ratio in the jejunum and ileum [33]. Regarding the underlying mechanism of the mitigative effect of baicalin on intestinal impairment, the authors demonstrated that baicalin suppressed the NF-κB pathway and activated the mammalian target of rapamycin (mTOR) signaling to regulate the downstream oxidative and inflammatory responses [33]. Overall, baicalin can efficiently scavenge free radicals in order to prevent oxidative injury. Additionally, baicalin has been reported to readily chelate with Cu^2+^ or Zn^2+^, which results in a metal chelate compound with a stronger free radical scavenging capacity than baicalin itself [36]. It has been found that dietary supplementation with 5 g/kg baicalin copper for 14 days markedly upregulated jejunal HO-1 protein expression of weaned piglets [36]. Moreover, baicalin copper supplementation notably elevated Mn-SOD activity and the concentrations of arginine, isoleucine, valine, lysine, and tyrosine in the serum of piglets fed DON-contaminated feed [36]. Baicalin zinc, a metal chelate compound with baicalin and Zn^2+^, has also been reported to possess potential antioxidant properties against DON-induced intestinal toxicity in piglets [49]. Specifically, dietary supplementation with 5 g/kg baicalin zinc for 14 days notably elevated serum T-AOC levels of piglets challenged with 4 mg/kg DON, suggesting that baicalin zinc can efficiently mitigate oxidative stress and enhance nutrient absorption in piglets fed DON-contaminated feed [49]. Hence, baicalin and its metal chelate compounds (baicalin copper and baicalin zinc) could be used as a feed additive for pigs against DON-induced intestinal toxicity.

### 4.4. Ferulic Acid

Ferulic acid (C_10_H_10_O_4_), a natural polyphenol substance, is widely found in fruits and vegetables such as rice grain, sweet corn, and tomatoes [50]. Ferulic acid is also a main bioactive compound of multiple Chinese herbal medicines, such as coptis and angelica [37]. Ferulic acid has been demonstrated to have multiple physiological functions, especially antioxidant and anti-inflammation properties [51]. Most recently, ferulic acid has been reported as a feed additive to improve the meat quality and carcass traits of finishing pigs [52,53], as well as to modulate antioxidant status and lipid metabolism in weaned piglets [54]. Li et al. (2015) reported that dietary supplementation with 100 mg/kg ferulic acid for 28 days elevated the pH_45 min_ value of the longissimus dorsi muscle and GSH-Px activity in the liver, and reduced MDA levels in the livers of finishing pigs [52]. Interestingly, Valenzuela-Grijalva et al. (2021) demonstrated that 25 mg/kg ferulic acid could be used as an alternative to ractopamine to promote the growth of finishing pigs [53]. The authors found that the dietary addition of 25 mg/kg ferulic acid for 27 days elevated the average daily gain and loin muscle area and the number of muscle fibers, and reduced fat deposition and the cross-sectional area of finishing pigs [53]. Wang et al. (2020) also documented that dietary inclusion of 0.45% ferulic acid for 35 days elevated the total superoxide dismutase (T-SOD) and catalase (CAT) activities, as well as the high-density liptein cholesterol (HDL-C) level, in serum. However, the levels of MDA, total cholesterol (T-CHO), and low-density lipoprotein (LDL-C) were reduced in the serum of weaned piglets [54]. Moreover, dietary ferulic acid supplementation improved the antioxidant status of the muscles and liver, and was found to be associated with activating the Nrf2 antioxidant signaling pathway and regulating the gene expression downstream of the Nrf2 pathway [54]. The protective effects of ferulic acid on porcine intestinal damage induced by DON exposure were confirmed in an in vitro model [37]. Ferulic acid treatment reversed the cell viability reduction in IPEC-J2 cells, which was caused by DON exposure [37]. The authors further investigated the underlying mechanism of the protective role of ferulic acid against DON, and found that the treatment of ferulic acid alleviated intracellular oxidative stress, inflammation, and apoptosis by regulating the Nrf2, MAPKs, and NF-κB pathways [37]. Herein, it is demonstrated that ferulic acid could be used as a feed additive suitable for pigs under DON exposure conditions.

### 4.5. Quercetin

Quercetin (C_15_H_10_O_7_), a flavonoid, is abundantly present in vegetables and fruits as a secondary antioxidant metabolite [38]. Quercetin has a strong antioxidant capacity, which gives it a perfect preventive and therapeutic effect on diseases caused by oxidative stress [55]. The antioxidant property of quercetin was previously proven in the porcine intestine in vitro and in vivo experiments. Zou et al. (2016) found that dietary supplementation with 25 mg/kg quercetin for 28 days alleviated intestinal integrity damage linked to reducing the intestinal oxidative stress and inflammation of finishing pigs subjected to transport stress [56]. Importantly, quercetin supplementation decreased the levels of ROS and MDA in the intestine, suggesting a beneficial role of quercetin in antioxidative stress of the intestines in pigs [56]. Meanwhile, the antioxidant status and meat quality of finishing pigs were markedly improved when pigs were fed a 25 mg/kg quercetin diet for 28 days, followed by a 5 h transport stress challenge [57]. Consistently, Xu et al. (2021) noted that dietary addition of 0.1% quercetin for 14 days improved intestinal antioxidant ability and alleviated diarrhea and weaning-associated intestinal injury in weanling piglets [58]. Specifically, supplementation of quercetin elevated the T-AOC, CAT level, and GSH/oxidized glutathione (GSSG) ratio, but reduced the MDA level in the jejunum of weanling piglets [58]. Moreover, quercetin supplementation reduced the fecal score and improved the intestinal barrier function of weanling piglets, as shown by elevated Occludin protein expression, villus height, villus height/crypt depth ratio, and reduced crypt depth and gut-epithelial apoptosis [58]. However, according to Degroote et al. (2019), short-term supplementation of quercetin for 14 days had little impact on the small intestine, but enhanced the glutathione transferase level of the liver in piglets [59]. In an IPEC-1 cell model, Jia et al. (2021) found that pretreatment with 5 μM quercetin for 24 h greatly abrogated diquat-induced intestinal damage, including cell apoptosis and oxidative stress, as indicated by increased ROS production and elevated mitochondrial depolarization [60]. Using a specific inhibitor of Nrf2 (all-trans-retinoic acid), the authors further demonstrated that the protective effect of quercetin on enterocytes was mediated via activating the Nrf2 signaling pathway and redox homeostasis [60]. Karancsi et al. (2022) reported the protective impact of quercetin and its methylated derivatives on inflammation in IPEC-J2 cells, acting against the lipopolysaccharide challenge [55]. A dosage of 50 μM quercetin or its methylated derivatives all decreased intracellular ROS levels, extracellular H_2_O_2_ levels, and IL-6 levels of IPEC-J2 cells challenged with lipopolysaccharide [55]. The aforementioned results highlight that quercetin could be used as a promising substance to protect the intestine against oxidative stress. Vergauwen et al. (2016) reported that quercetin was beneficial at a concentration range of 25–800 mmol/L to reduce intracellular ROS levels and strengthen the integrity of the monolayer of IPEC-J2 cells [61]. Similarly, Chen et al. (2018) also found that quercetin treatment promoted cell proliferation, and protected cells from H_2_O_2_-induced apoptosis in IPEC-J2 cells [62]. Specifically, quercetin incubation decreased the apoptosis rate, Bax/Bcl-2 ratio, and ROS level, but increased mitochondrial membrane potential in IPEC-J2 cells subjected to an H_2_O_2_ challenge [62]. Most importantly, Pomothy et al. (2021) demonstrated that pretreatment with quercetin protected against DON-induced monolayer integrity damage and oxidative stress in IPEC-J2 cells [38]. It is suggested that quercetin might scavenge ROS in two ways. One mechanism can be that quercetin directly acts on both intracellular superoxide anion radicals or other free radicals and eliminates them [38]. The other possible mode of action of quercetin seems to involve the initiation of antioxidant pathways in cells via promoting the production of antioxidant enzymes [38]. In vivo feeding trials are needed to confirm the beneficial effects of quercetin against DON-induced intestinal toxicity in piglets, and the optimum supplementation dosage has yet to be confirmed.

### 4.6. Dihydromyricetin

Dihydromyricetin (C_15_H_12_O_8_) is a natural flavonoid originating from the Ampelopsis grossedentata plant, and has been demonstrated to have strong antioxidant and anti-inflammatory characteristics [29,63,64]. Most recently, dihydromyricetin has been demonstrated to promote the intestinal antioxidant ability of pigs in vivo [65]. Concretely, feeding 0.03% dihydromyricetin to grower pigs for 15 weeks markedly enhanced T-AOC and the activities of GSH-Px and CAT, but reduced MDA content in the jejunum mucosal of pigs [65]. More importantly, dihydromyricetin has been reported to have protective effects against DON-induced toxicity in porcine intestinal epithelial cells [29]. Compared with the DON treatment group, cotreatment with dihydromyricetin markedly promoted cell viability and inhibited cell apoptosis, and improved antioxidant capacity and anti-inflammatory ability, as evidenced by the increased intracellular GSH content, and decreased intracellular ROS, tumor necrosis factor-α (TNF-α), and interleukin-8 (IL-8) levels [29]. The results of Long et al. (2021) indicate that dihydromyricetin can effectively alleviate the DON-induced toxicity of porcine intestinal epithelial cells by reducing intestinal oxidative stress, inflammation, and apoptosis [29]. Additionally, dihydromyricetin has also been reported to improve intestinal barrier function, as well as nutrient transport and absorption in pigs [66]. Wei et al. (2022a) found that dietary dihydromyricetin supplementation significantly upregulated the mRNA expression of MUC1 and MUC2 and the protein expression of Claudin-1 and Occludin in the jejunum mucosa of pigs [66]. Dietary dihydromyricetin supplementation also elevated the activities of maltase, lipase, amylase, and sucrase, and upregulated the mRNA expression of nutrient transporters (SGLT1, GLUT2, and PepT1) in the jejunum mucosa of pigs [66]. Therefore, the above research results suggest that the application of dihydromyricetin as a feed additive in pig diets has broad prospects, and it may feasibly be used to alleviate the toxic effects of feed DON contamination on pigs.

### 4.7. Chlorogenic Acid

Chlorogenic acid (C_16_H_18_O_9_) is a polyphenol compound extracted from vegetables, fruits, and plants, including Eucommia ulmoides, honeysuckle, coffee and tea, and so on [39]. In recent years, the beneficial regulation of chlorogenic acid on the intestinal function and health of pigs has attracted widespread attention. The primary physiological and biochemical function of chlorogenic acid is attributed to its robust antioxidant function [67]. It has been reported that feeding 1000 mg/kg chlorogenic acid to weaned piglets for 2 weeks increased the activities of GSH-Px and CAT, and decreased MDA content in the duodenal and jejunal mucosa of pigs [68]. Meanwhile, the activities of GSH-Px, SOD, and CAT in the serum of piglets were elevated by dietary 1000 mg/kg chlorogenic acid supplementation [69]. These results suggest that dietary chlorogenic acid supplementation improved the antioxidant status of piglets, which is beneficial for piglets to protect against oxidative stress. Chen et al. (2022) found that the dietary addition of 1000 mg/kg chlorogenic acid for 21 days increased antioxidant enzyme activity in the serum of piglets under oxidative stress conditions [70]. Moreover, dietary supplementation with 1000 mg/kg chlorogenic acid for 2 weeks markedly decreased the mRNA expression of IL-6, TNF-α, IL-1β, and NF-κB in the jejunal and ileal mucosa of piglets [71]. Regarding intestinal apoptosis, chlorogenic acid supplementation decreased apoptotic cells in the jejunum as well as Bax/Bcl-2 ratio in the duodenum and jejunum of piglets [71]. It has been reported that feeding 1000 mg/kg chlorogenic acid to weaned piglets for 2 weeks enhanced the intestinal barrier function of piglets, as indicated by decreased serum diamine oxidase (DAO) and D-lactate levels, and upregulated protein expression of claudin-1 in the small intestinal epithelium [68]. Those results suggest that chlorogenic acid improved intestinal barrier function and reduced intestinal oxidative stress, inflammation, and apoptosis of piglets. In an IPEC-J2 cell model, pre-treatment with 40 μg/mL chlorogenic acid for 1 h elevated cell viability and reduced LDH release and apoptosis in IPEC-J2 cells, which were challenged by 0.5 μg/mL DON for 6 h in comparison to the DON-treated IPEC-J2 cells [39]. Under DON exposure conditions, chlorogenic acid supplementation still exerts robust protective effects on porcine intestinal epithelial cells, including maintaining intestinal barrier function and nutrient transport [39]. Concretely, the mRNA expression of Claudin-1, ZO-1, Occludin, PePT1, and GLUT2 were upregulated when IPEC-J2 cells were treated with chlorogenic acid under DON exposure conditions [39]. This study indicated that chlorogenic acid could alleviate porcine enterotoxicity induced by DON exposure via its antioxidant, anti-inflammation, and anti-apoptosis characteristics [39]. Therefore, chlorogenic acid is a promising polyphenol to counteract the oxidative damage and intestinal injury of swine by DON exposure.

### 4.8. Astilbin

Astilbin (C_21_H_22_O_11_), a natural plant-derived flavonoid polyphenol compound, was isolated from Hypericum perforatum [72], Engelhardtia chrysolepis, and other plants [73]. Astilbin has been reported to possess antioxidant, anti-inflammatory, anti-microbial, and immune-regulatory properties [28]. Nakahara et al. (2017) reported that astilbin improved intestinal barrier functions in Caco-2 cells [73]. Specifically, astilbin treatment elevated the transepithelial electrical resistance and the relative mRNA levels of ZO-2 and Claudin-1, as well as the protein expression of Occludin and ZO-2 in Caco-2 cells [73]. Xu et al. (2020) found that 0.5 μg/mL DON exposure for 6 h led to cellular oxidative stress, inflammation, and cell apoptosis in IPEC-J2 cells [28]. However, compared with DON exposure, treatment of IPEC-J2 cells with 20 μg/mL astilbin significantly improved cellular oxidative stress status, as indicated by decreased MDA content, as well as the increased CAT and SOD activities [28]. Additionally, 20 μg/mL astilbin treatment downregulated the relative mRNA expression of Bax, Bcl-2, and Caspase3, and increased the Bcl-2/Bax ratio, suggesting that astilbin reduced cell apoptosis caused by DON exposure [28]. Simultaneously, the cellular inflammation induced by DON was reduced by 20 μg/mL astilbin treatment, as reflected by the relative mRNA expression of IL-6, IL-8, TNF-α, COX-2, and NF-κB in IPEC-J2 cells [28]. Astilbin is another promising polyphenol to be utilized as a feed additive for pigs under DON exposure.

### 4.9. Rosmarinic Acid

Rosmarinic acid (C_18_H_16_O_8_) is a non-flavonoid phenolic compound. Vergauwen et al. (2016) concluded that pre-incubation of cells with 200, 400, and 600 μmol/L rosmarinic acid for 18 h could reinforce the IPEC-J2 cell monolayer integrity after the peroxide challenge [61]. According to Li et al. (2020), a chlorogenic acid-enriched extract derived from Eucommia ulmoides Oliver leaves (1000 mg/kg) improved the growth performance of finishing pigs, including average daily gain and average daily feed intake [74]. More importantly, 1000 mg/kg chlorogenic acid-enriched extract enhanced muscle antioxidant capacity, as indicated by increased T-AOC, GSH-Px, T-SOD, and sarcoplasmic reticulum Ca^2+^-ATPase activities, as well as upregulated SOD1 gene expression [74]. Additionally, the meat quality was improved by dietary supplementation of chlorogenic acid-enriched extract, as suggested by reduced cooking loss, drip loss, carbonyl, and MDA contents in the meat of pigs [74]. Using an in vitro model of IPEC-J2 cells, Pomothy et al. (2020) evaluated the impact of rosmarinic acid on intestinal oxidative stress and inflammation of porcine enterocytes induced by DON and T-2 toxin [30]. The authors found that a dosage of 50 μmol/L rosmarinic acid restored the decreased transepithelial electrical resistance of IPEC-J2 cell monolayer caused by cotreatment with 1 μmol/L DON + 5 nmol/L T-2 toxin for 48 or 72 h [30]. It was also reported that cotreatment with DON and T-2 toxin for 48 or 72 h markedly resulted in oxidative stress and increased interleukin-6 (IL-6) and IL-8 levels, which were mitigated by rosmarinic acid treatment [30]. These data indicate that rosmarinic acid administration is beneficial for defense against DON, T-2 toxin-induced cell monolayer integrity impairment, and the disturbed redox status of IPEC-J2 cells [30].

## 5. Potential Mechanism of Action: Activation of Nrf2 by the Polyphenols

**Figure 2:**
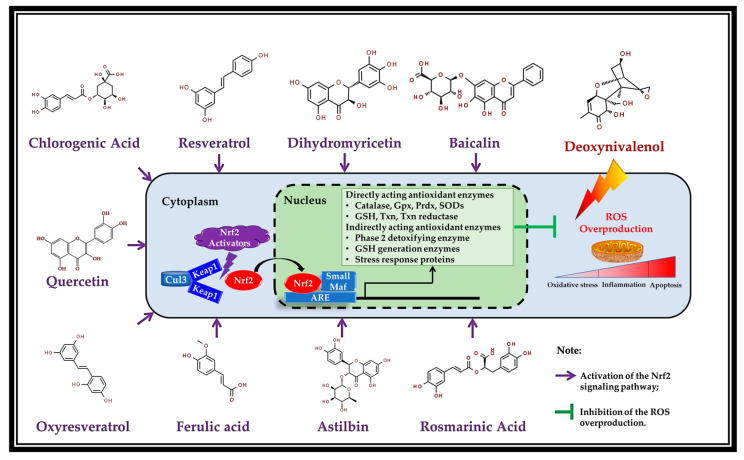
Activation of the Nrf2 pathway by the polyphenols (Resveratrol [10], dihydromyricetin [65], baicalin [79], chlorogenic acid [68], rosmarinic acid [80], quercetin [60], astilbin [81], oxyresveratrol [82], and ferulic acid [37]) (adapted from the reference [78]). Abbreviations: Gpx, glutathione peroxidase; GSH, glutathione; Prdx, peroxiredoxins; ROS, reactive oxygen species; SODs, superoxide dismutases; Txn, thioredoxin.

The Keap1-Nrf2-ARE pathway is the most critical endogenous antioxidant signaling pathway found in animal organisms, and is also the primary response element of cells to mycotoxin toxicity [75]. It has been demonstrated that the Keap1-Nrf2-ARE pathway can regulate more than 100 downstream genes, among which are redox signaling, antioxidant stress, and related target genes, with cell protection functions as the main ones [76]. The Keap1-Nrf2-ARE pathway mainly includes three functional parts: Keap1 (Kelch-like epichlorohydrin associated protein 1), Nrf2 (nuclear factor erythroid 2-like 2), and ARE (antioxidant response element), among which Nrf2 is a key regulator. Nrf2 is a redox-sensitive transcription factor which plays a vital role in modulating the cellular physiological responses to electrophilic and oxidative stress. It accomplishes this through modulating diverse downstream target genes encoding phase II detoxifying enzymes and antioxidant proteins [77]. Under normal conditions, Nrf2 is coupled to its repressor protein (Keap1) and exists in the cytoplasm in an inactive form. The Keap1 protein is anchored by cytosolic actin and acts as a substrate, which mediates the ubiquitination and degradation of Nrf2 through the Keap1-Cul3 ubiquitin ligase pathway, and maintains the Nrf2 protein in cells at a relatively low concentration. In the presence of Nrf2 activators (such as polyphenols), the binding of Keap1-Nrf2 interferes (see Figure 2). Cysteine in the IVR region of Keap1 is oxidized, leading to the change in its protein conformation. The DC-DLG binding site is broken, and uncouples with Nrf2. The dissociated Nrf2 protein is transferred to the nucleus to bind to Maf protein and ARE elements, thus driving the expression of downstream antioxidant enzymes and phase II detoxification enzyme genes [78].

There is an increasing body of shreds of evidence that plant-derived polyphenols are natural activators of Nrf2, can inhibit oxidative stress and inflammation via targeting Nrf2, and consequently driving ARE-associated antioxidant genes (Figure 2) [77]. Most recently, resveratrol has been demonstrated to alleviate H_2_O_2_-caused oxidative stress in IPEC-J2 through PI3K (phosphoinositide 3-kinase)/Akt (serine/threonine kinase)-mediated Nrf2 signaling pathway [83]. Wei et al. (2022) reported that dietary dihydromyricetin supplementation upregulated the protein expression of nuclear Nrf2, HO-1, and NQO1 in the jejunum of growing-finishing pigs, suggesting that dihydromyricetin supplementation activated Nrf2 and drives its downstream antioxidant target genes [65]. The authors also found that dihydromyricetin treatment promoted the nuclear translocation of Nrf2 and the protein expression of HO-1 in vitro using an IPEC-J2 cell model [65]. In addition, Bao et al. (2021) noted that baicalin remitted oxidative injury of IPEC-J2 cells subjected to a lipopolysaccharide challenge, activated the Nrf2 pathway, and upregulated the mRNA and protein levels of its downstream antioxidant protein, such as HO-1 (heme oxygenase-1) and NQO-1 (quinone oxidoreductase-1) [79]. Similarly, quercetin has been proven to restore diquat-induced oxidative injury via activating Nrf2 signaling in IPEC-1 cells [60]. In weaned piglets, the dietary addition of chlorogenic acid elevated the protein expressions of Nrf2 and HO-1 in the duodenal and jejunal mucosa, indicating that the Nrf2 pathway was activated and its downstream HO-1 expression was driven by chlorogenic acid [68]. Furthermore, the Nrf2 pathway has been also demonstrated to be activated by rosmarinic acid in laying hens [80], astilbin in HEK-293 cells [81], and oxyresveratrol in HepG2 cells [82]. Most importantly, Yang et al. (2019) documented that treatment with resveratrol effectively activated the Nrf2 signaling pathway and promoted antioxidant protection against DON-induced toxicity in IPEC-J2 cells. The authors further confirmed the essential role of Nrf2 via Nrf2 siRNA knockdown, and found that Nrf2 siRNA knockdown abrogated the protective effects of resveratrol against DON-caused intestinal cytotoxicity [10]. Meng et al. (2022) found that ferulic acid ameliorated DON-induced oxidative stress and cellular toxicity, and activated the Nrf2 pathway in IPEC-J2 cells, as suggested by the downregulated protein expressions of Keap1 and cytoplasm Nrf2, as well as the upregulated protein of nuclear Nrf2 [37]. As the downstream target antioxidant gene of Nrf2, HO-1 was also upregulated at the protein level by ferulic acid treatment in IPEC-J2 cells [37]. Therefore, the Nrf2 regulation by polyphenols is supposed to be the potential underlying mechanism of action of polyphenols in protecting against DON-induced enterotoxicity of swine. In light of the increasing evidence that multiple polyphenols share a joint pharmacological mechanism as Nrf2 activators, this extensive group of substances provides a batch of candidates for treating oxidative stress and intestinal damage of swine exposure to DON contamination.

## 6. Conclusions and Perspectives

In this review, we systematically summarized various studies available in terms of plant-derived polyphenols as Nrf2 activators ameliorating DON-induced oxidative stress and intestinal toxicity in swine. These studies demonstrated that polyphenols can counteract the deleterious effects of chronic consumption of DON, and confirmed the potential effectiveness of dietary strategies to counteract DON-induced intestinal toxicity in pigs. To date, although only nine polyphenols have been reported to activate Nrf2 and mitigate DON-caused enterotoxicity of swine in vitro and/or in vitro, more than 8000 polyphenol compounds derived from plants have been identified. There a is much room to explore more polyphenols (or phytogenic extracts rich in polyphenols) as functional feed additives in swine. Furthermore, activation of the porcine intestinal Nrf2 pathway can be utilized as a key criterion (marker) for screening potential high-efficiency plant polyphenol feed additives for swine, especially under feed DON contamination conditions. In addition, further studies are warranted to evaluate the additive effects of screened polyphenols under feeding trial conditions, and to determine the optimal application dosage of polyphenols or polyphenol-enriched plant extracts at different production stages of swine. In light of the current findings, the development and application of polyphenols is an emerging and promising direction for future investigation in this research field.

## Figures and Tables

**Table 1:** The summary of plant-derived polyphenols counteracting deoxynivalenol (DON)-induced enterotoxicity in swine in vivo or in vitro.

Polyphenols	In Vivo/In Vitro Model	Polyphenol Doses and Duration	DON Doses and Duration	Main Findings (Polyphenol + DON Treatment vs. DON Treatment)	References
Resveratrol	Piglets	300 mg/kg, 21 days (Cotreatment with DON)	2.65 mg/kg, 21 days	Improved jejunal morphology (↑ villi height, villi height/crypt depth)	[21]
				Promoted antioxidant capacity of jejunum mucosa (↑ SOD, T-AOC, ↓ MDA)	
				Reduced oxidative stress of jejunum (↓ mitochondria ROS level, ↑ mitochondrial membrane potential)	
Resveratrol	Piglets	300 mg/kg, 28 days (Cotreatment with DON)	3.8 mg/kg, 28 days	Improved intestinal barrier function (↓ plasma D-lactase level, ↑ ZO-1 mRNA and protein expression in jejunal mucosa)	[31]
				Decreased intestinal inflammation: (↓ TNF-α and IL-1β mRNA and protein expression in jejunal mucosa)	
				Improved antioxidant capacity of the jejunum (↑ GCLC, SOD1, GCLM, NQO-1, and HO-1 mRNA expression, ↑ GCLM protein expression, ↓ MDA level)	
				Alleviated intestinal apoptosis (↓ TUNEL-positive cells percentage, caspase3 protein expression)	
				Beneficially regulated colon microbiota (↑ Roseburia and butyrate, ↓ Bacteroides and unidentified-Enterobacteriaceae)	
Resveratrol	Piglets	300 mg/kg, 28 days (Cotreatment with DON)	3.8 mg/kg, 28 days	Decreased ileum mitophagy: (↓ PINK1, Parkin, Beclin-1, Lamp, Atg5, Map1lc, Bnip3, Fundc1, Bcl2l1 and SQSTMS1)	[34]
				Improved mitochondrial functions (↑ SDHA, PHB1, and VDAC protein expression)	
Resveratrol	IPEC-J2 cells	15 µM, 24 h (pretreatment)	0.5 μg/mL, 24 h	Enhanced the rate of the spare respiratory capacity of IPEC-J2 cells	[34]
				Elevated the total ATP and NAD of IPEC-J2 cells	
Resveratrol	IPEC-J2 cells	15 µM, 24 h (pretreatment)	0.5 μg/mL, 24 h	Improved cell viability and cell proliferation	[10]
				Decreased oxidative stress and apoptosis (↓ intracellular ROS, apoptotic cells, ↑ mitochondrial membrane potential)	
				Activated Nrf2 pathway	
Resveratrol	IPEC-J2 cells	50 µM, 12 h (1 h pretreatment and 11 h cotreatment with DON)	4 μM, 11 h	Contributed to maintaining cell monolayer integrity (↑ TEER)	[32]
				Improved intestinal barrier function (↑ mRNA expression: Claudin 3 and Claudin 4; ↑ protein expression: Claudin-4)	
				Decreased cellular inflammation (↓ IL-8, IL-6)	
				Inhibited MAPKs pathway (↓ p-JNK, p-ERK, p-p38)	
Oxyresveratrol	IPEC-J2 cells	25 µM, 12 (1 h pretreatment and 11 h cotreatment with DON)	4 μM, 12 h	Improved intestinal barrier function (↑ protein expression: Claudin-4; ↑ TEER)	[35]
				Inhibited JNK MAPK pathway (↓ p-JNK)	
Baicalin	Piglets	0.1%, 14 days (Cotreatment with DON)	4 mg/kg, 14 days	Improved growth performance (↑ ADFI, ADG, ↓ F/G)	[33]
				Alleviated the disorder of serum biochemical indices (↓ ALP, ALT, AST, LDH)	
				Decreased inflammatory response (↓ serum level: IL-8, IL-1β, IL-6, IFN-γ, TNF-α; ↓ Ileal mRNA expression: IL-8, IL-1β, IL-6, IFN-γ, TNF-α; ↓ jejunal mRNA expression: IL-8, IFN-γ, TNF-α)	
				Improved serum antioxidant capacity (↑ T-AOC, GSH-Px, GSH, SOD; ↓ MDA)	
				Improved intestinal morphology (↑ ileum and jejunum: villus height and villus height/crypt depth)	
				Inhibited NF-κB pathway (↓ ileal and jejunal gene expression and protein levels: NF-κB p65)	
				Activated mTOR pathway (↑ ileal and jejunal gene expression and protein levels: mTOR)	
Baicalin-copper	Piglets	5 g/kg, 14 days (Cotreatment with DON)	4 mg/kg, 14 days	Increased intestinal absorption of amino acids (↑ serum level: Lys, Val, Tyr, Ile, Leu, and Arg)	[36]
Ferulic acid	IPEC-J2 cells	60 µM, 12 h (pretreatment)	40 µM, 12 h	Increased cell viability	[37]
				Decreased cellular oxidative stress: (↓ intracellular ROS, ↑ SOD and GSH)	
				Activated Keap1/Nrf2 pathway and upregulated protein expression of the downstream antioxidant protein (↑ nuclear Nrf2, HO1, ↓ cytoplasm Nrf2, keap1)	
				Decreased cellular inflammation (↓ IL-6 and IL-8)	
				Decreased the phosphorylation level of MAPKs (↓ p-p38/p38, p-JNK/JNK, and p-ERK1/2/ERK1/2)	
				Inhibited the activation of NF-κB pathway (↓ p-IκB-α/IκB-α)	
				Attenuated the DON-induced intracellular apoptosis (↑ Bcl-2, ↓ Bax, caspase-3)	
Quercetin	IPEC-J2 cells	20 μM, 24 h (pretreatment)	1 μM, 1 h	Pretreatment with quercetin contributed to maintaining cell monolayers integrity (↑ TEER)	[38]
				Pretreatment with quercetin alleviated cellular oxidative stress (↓ H_2_O_2_, ROS)	
Dihydromyricetin	IPEC-J2 cells	40 μM, 24 h (cotreatment with DON)	250 ng/mL, 24 h	Decreased the cytotoxicity caused by DON (↑ cell viability, ↓ apoptotic cells)	[29]
				Decreased cellular oxidative stress (↓ intracellular ROS; ↑ intracellular GSH)	
				Decreased cellular inflammation (↓ culture supernatants concentration: TNF-α, IL-8; ↓ mRNA expression: TNF-α, IL-6, IL-8, IL-1β)	
				Restored the metabolism disorders of metabolic pathways including histidine metabolism, glutamate metabolism, and arachidonic metabolism	
Chlorogenic acid	IPEC-J2 cells	40 µg/mL, 1 h (Pretreatment)	0.5 µg/mL, 6 h	Decreased the cytotoxicity caused by DON (↓ LDH)	[39]
				Decreased cellular inflammation (↓ mRNA expression: TNF-α, IL-6, IL-8, COX-2; ↓ protein expression: COX-2)	
				Decreased cell apoptosis (↓ cell apoptosis rate; ↓ mRNA expression: caspase-3, Bax; ↓ protein expression: Bax)	
				Increased barrier function (↑ mRNA expression: ZO-1, Occludin, Claudin-1; ↑ protein expression: ZO-1, Claudin-1)	
				Increased nutrient transport (↑ mRNA expression: PePT1 and GLUT2; ↑ protein expression: PePT1)	
Astilbin	IPEC-J2 cells	20 μg/mL, 6 h (cotreatment with DON)	0.5 μg/mL, 6 h	Decreased the cytotoxicity induced by DON (↑ cell viability, ↓ LDH)	[28]
				Increased cellular antioxidant capacity (↑ CAT, SOD, ↓ MDA)	
				Decreased cellular inflammation (↓ mRNA expression: TNF-α, IL-6, IL-8, COX-2, and NF-κB)	
				Decreased cell apoptosis (↓ cell apoptosis rate; ↓ mRNA expression: Bax, Caspase3; ↑ Bcl-2/Bax)	
				Increased barrier function (↑ mRNA expression: ZO-1, Claudin-1)	
				Increased nutrient transport (↑ mRNA expression: PePT1).	
Rosmarinic acid	IPEC-J2 cells	50 μM, 24 h (pretreatment)	1 μM DON and 5 nmol/L T-2, 48 and 72 h	Cotreatment with DON and T-2 reduced TEER of the cell monolayer, which was restored by rosmarinic acid;	[30]
				Cotreatment with DON and T-2 induced oxidative stress and elevated IL-6 and IL-8 levels, which were alleviated by rosmarinic acid.	

↑ = increase; ↓ = decrease; ADFI = average daily feed intake; ADG = average daily gain; ALP = alkaline phosphatase; ALT = alanine aminotransferase; AST = aspartate aminotransferase; ATP = adenosine triphosphate; Bax = Bcl-2 associated x protein; Bcl-2 = B-cell lymphoma/leukemia 2; COX-2 = cyclooxygenase-2; F/G = the ratio of feed to gain; GCLC = glutamate cysteine ligase catalytic subunit; GCLM = glutamatecysteine-ligase modulatory subunit; GLUT2 = glucose transporter 2; GSH = glutathione; GSH-Px = glutathione peroxidase; HO-1 = heme oxygenase-1; IFN-γ = interferon γ; IL-1β = interleukin-1β; IL-6 = interleukin-6; IL-8 = interleukin-8; JNK = c-Jun N-terminal kinase; keap1 = kelch-like epichlorohydrin associated protein 1; LDH = lactate dehydrogenase; MDA = malondialdehyde; mTOR = mammalian target of rapamycin; NAD = nicotinamide adenine dinucleotide; NF-κB = nuclear factor kappa-B; NQO-1 = NAD(P)H dehydrogenase, quinone 1; Nrf2 = nuclear factor erythroid 2-like 2; PePT1 = peptide transporter 1; PINK1 = PTEN-induced kinase 1; ROS = reactive oxygen species; SOD = superoxide dismutase; SOD1 = superoxide dismutase 1; T-AOC = total antioxidant capacity; TEER = transepithelial electrical resistance; TNF-α = tumor necrosis factor-α; TUNEL = terminal deoxynucleotidyl transferase mediated dUTP nick end labeling; ZO-1 = zonula occludens-1.

## Data Availability

Not applicable.
